# Microbiology and epidemiology of enteroaggregative Escherichia coli isolated from UK residents in England, 2016–2023: what are the risks to public health?

**DOI:** 10.1099/jmm.0.002097

**Published:** 2025-11-13

**Authors:** Ching-Ying J. Poh, Ella V. Rodwell, David R. Greig, Satheesh Nair, Marie A. Chattaway, Claire Jenkins

**Affiliations:** 1 Gastrointestinal Bacteria Reference Unit, UK Health Security Agency, London NW9 5HT, UK; 2 National Institute for Health and Care Research Health Protection Research Unit in Gastrointestinal Infections, University of East Anglia, Norwich NR4 7TJ, UK; 3 Gastrointestinal Infections & Food Safety (One Health), UK Health Security Agency, London, UK

**Keywords:** antimicrobial resistance, enteroaggregative *Escherichia coli*, molecular diagnostics, public health, surveillance, travellers' diarrhoea

## Abstract

**Introduction.** Following two large foodborne outbreaks of the gastrointestinal pathogen, enteroaggregative *Escherichia coli* (EAEC), in Germany in 2011 and the UK in 2014, the UK Health Security Agency (UKHSA) implemented enhanced surveillance strategies for EAEC.

**Gap Statement.** The surveillance of diarrhoeagenic *E. coli* in England focuses on Shiga toxin-producing *E. coli* (STEC), and the true clinical and community burden of EAEC is unknown. This gap extends globally, as many countries lack the infrastructure, diagnostic tools and healthcare facilities to resource surveillance programmes for EAEC.

**Aim.** The aim of the study was to review the microbiological typing data and demographic data linked to isolates and cases diagnosed from 2016 to 2023 and to assess the risk to public health.

**Methodology.** Faecal samples that tested positive by PCR for diarrhoeagenic *E. coli* at local microbiology diagnostic laboratories were referred to the UKHSA for confirmation and culture. Isolates identified as EAEC were sequenced on the Illumina HiSeq and NextSeq platforms. Sequence type, serotype and antimicrobial resistance (AMR) profile were derived from the genome sequence. Age, sex and travel histories were linked to the typing data.

**Results.** There was a total of 1,402 notifications of EAEC, exhibiting a fivefold increase in diagnoses from 93 in 2016 to 524 in 2023. The most common sequence types (STs) were ST34 (*n*=202/1,402, 14.4%), ST10 (*n*=185/1,402, 13.2%), ST200 (*n*=183/1,402, 13.1%) and ST678 (*n*=101/1,402, 7.2%), and the most common serotypes were O92:H33 (*n*=130/1,402, 9.3%), O175:H31 (*n*=78/1,402, 5.6%) and O99:H10 (*n*=78/1,402, 5.6%). Most cases were female (*n*=748/1,372, 54.5%) and/or were aged <10 (*n*=387/1,372, 28.2%), within which 299 out of 387 (77.3%) were <5 years old. Of the 756 out of 1,386 (54%) cases that had a travel history, 597 out of 756 (79%) reported foreign travel within 7 days of onset of symptoms. AMR was detected in 1,030 out of 1,402 (73.5%) isolates with resistance to fluoroquinolone (*n*=810/1,402, 57.8%) and beta-lactam (*n*=807/1,402, 57.6%) antibiotics being the most common.

**Conclusion.** Given the burden of disease caused by EAEC in the community, the high proportion of infections in children and travellers, the risk of the emergence of hybrid STEC/EAEC pathotypes and the high proportion of AMR, we recommend that EAEC should be part of the diagnostic algorithm in the UK.

## Introduction

Enteroaggregative *Escherichia coli* (EAEC) are gastrointestinal pathogens that cause severe abdominal pain and profuse, persistent diarrhoea, most commonly in children and travellers returning from low- or middle-income countries [1,6]. Although often prolonged (lasting for 10 days or more), symptoms are generally self-limiting in healthy individuals. However, in vulnerable groups, such as malnourished children or adults with HIV, infection can be debilitating [1,5,7]. Global infectious intestinal disease (IID) studies indicate that EAEC are a common cause of symptoms of gastrointestinal disease worldwide, with a particularly high prevalence of EAEC in Africa and Asia [8,11].

The EAEC belong to a diverse group of pathogens harbouring a variety of different plasmid and chromosomally encoded virulence factors, including genes implicated in aggregative adherence fimbriae biogenesis and toxin production [12,15]. Several toxins have been linked to EAEC virulence, including *Shigella* enterotoxin 1, plasmid‐encoded toxin and EAST-1 [16]. Strains of EAEC are defined by the presence of *aggR*, a gene encoding a 794 bp protein (AggR), exhibiting a high degree of amino acid sequence identity to the AraC class of gene regulators, that operates as a transcriptional activator of fimbrial expression [16,17]. The fimbriae produced by EAEC mediate adherence to the human gut mucosa in a characteristic stacked brick pattern and promote colonization and infection [18, 19].

Transmission is most likely driven by person-to-person contact, similar to *Shigella* species. EAEC has been associated with foodborne outbreaks, although, like *Shigella*, contamination may be caused by human faeces rather than animal faeces [20,25]. There are anecdotal reports of EAEC being isolated from animal faeces; however, these findings may represent transient carriage rather than established colonization. The evidence for EAEC being a classic zoonotic pathogen with a well-defined animal reservoir remains unclear [26,28].

Most *E. coli* virulence factors are encoded on mobile genetic elements, and hybrid strains harbouring virulence genes from different pathotypes can occur. The most infamous foodborne outbreaks in recent times occurred in Germany in 2011, caused by contaminated fenugreek seeds [29,31]. The aetiological agent was a typical strain of EAEC that acquired a bacteriophage encoding Shiga toxin, resulting in a highly pathogenic STEC-EAEC combination that caused over 900 cases of Haemolytic uraemic syndrome (HUS) and 54 deaths [30]. The emergence of extraintestinal *E. coli* harbouring *aggR* has also been described [32].

In the aftermath of the outbreak of *E. coli* O104 in Germany, there was heightened awareness of the risk of emerging pathogenic variants of *E. coli*, and many countries, including the UK, stepped up their surveillance of EAEC [33]. Although sporadic cases of hybrid STEC-EAEC continue to be detected, there have been no further outbreaks documented to date [34,35]. We reviewed the microbiology and epidemiology of EAEC isolates from UK residents in England from January 2016 to December 2023. The aim of this study was to use the available data to assess the risk to public health and to inform future testing and surveillance strategies for EAEC in the UK.

## Methods

### Microbiology

Clinical infection is diagnosed by detecting EAEC in faecal specimens using PCR targeting *aggR* [16]. Although EAEC are not included in the local hospital diagnostic microbiology testing algorithm in the UK, faecal specimens submitted to the Gastrointestinal Bacteria Reference Unit (GBRU), UK Health Security Agency (UKHSA), for testing for Shiga toxin-producing *E. coli* (STEC) are concurrently tested for EAEC, in addition to enteroinvasive (EIEC), enterotoxigenic (ETEC) and enteropathogenic *E. coli* (EPEC) [2].

All faecal specimens submitted to GBRU were inoculated onto MacConkey agar and into tryptone soya broth (TSB) and incubated at 37 °C. Following overnight incubation, DNA was extracted from TSB using InstaGene (Bio-Rad catalogue no. 732–6030) and tested using the EU-RL VTEC real-time PCR primers and probes detecting *stx*, *aggR*, *ipaH*, *lt/st* and *eae*, defining virulence genes for STEC, EAEC, EIEC, ETEC and EPEC, respectively, on a Rotor-Gene Q (Qiagen, UK) as described previously [2]. For all faecal specimens that tested positive for *stx*, ten colonies were selected from the MacConkey agar and retested using the same PCR.

Microbiological results are stored in the Gastro Data Warehouse (GDW), an in-house UKHSA database for storing and linking patient demographic and microbiological typing data.

### Epidemiological data

Patient information, including sex, age and recent travel, was collected from laboratory request forms upon submission and stored in GDW.

### DNA extraction, genome sequencing and quality control

Genomic DNA from all isolates was extracted using the QIAsymphony (Qiagen). The sequence library was prepared using the Nextera XT DNA and Nextera Flex sample preparation kits (Illumina) and sequenced using the Illumina HiSeq 2500 and NextSeq 1000 platforms (100 bp paired-end reads) at UKHSA. Trimmomatic (v0.32) was used to trim sequence adapters. Reads were discarded if they fell below a PHRED score of 30 from the leading and trailing ends, or if the read length was less than 50 bp.

FASTQ reads from all sequences in this study can be found at the UKHSA Pathogens BioProject at the National Center for Biotechnology Information (BioProject number PRJNA315192) (Table S1, available in the online Supplementary Material).

### Sequence typing

Sequence type (ST) assignment was performed using a modified version of short-read sequence typing (SRST) using the MLST (v2.15–2.17) database described by Tewolde *et al.* [36]. The MOST software (for MLST) is available at https://github.com/ukhsa-collaboration/MOST. All scripts listed were run using default parameters. GrapeTree was employed to generate a minimum spanning tree (MSTree-V2) [37]. This was visualized in the GrapeTree platform and annotated with ST derived from SRST2. Where there was a probable ST (due to the database not holding all the allelic variants), this was checked in EnteroBase [38].

### Virulence and AMR profiling

Virulence profiling was performed via *GeneFinder* (v2.9–2.11) (https://github.com/ukhsa-collaboration/gene_finder) [2]. Genes were confirmed to be present if the coverage and sequence similarity were over 85% compared to the reference gene. Antimicrobial resistance (AMR) profiling was performed, and the presence of AMR genes was determined using *GeneFinder* (https://github.com/ukhsa-collaboration/gene_finder) [3]. AMR profiles are displayed using UpSetR (https://hidivelab.org/research/projects/upsetr/).

## Results

### Overview of the dataset: sequence typing and serotyping

**Fig. 1.**
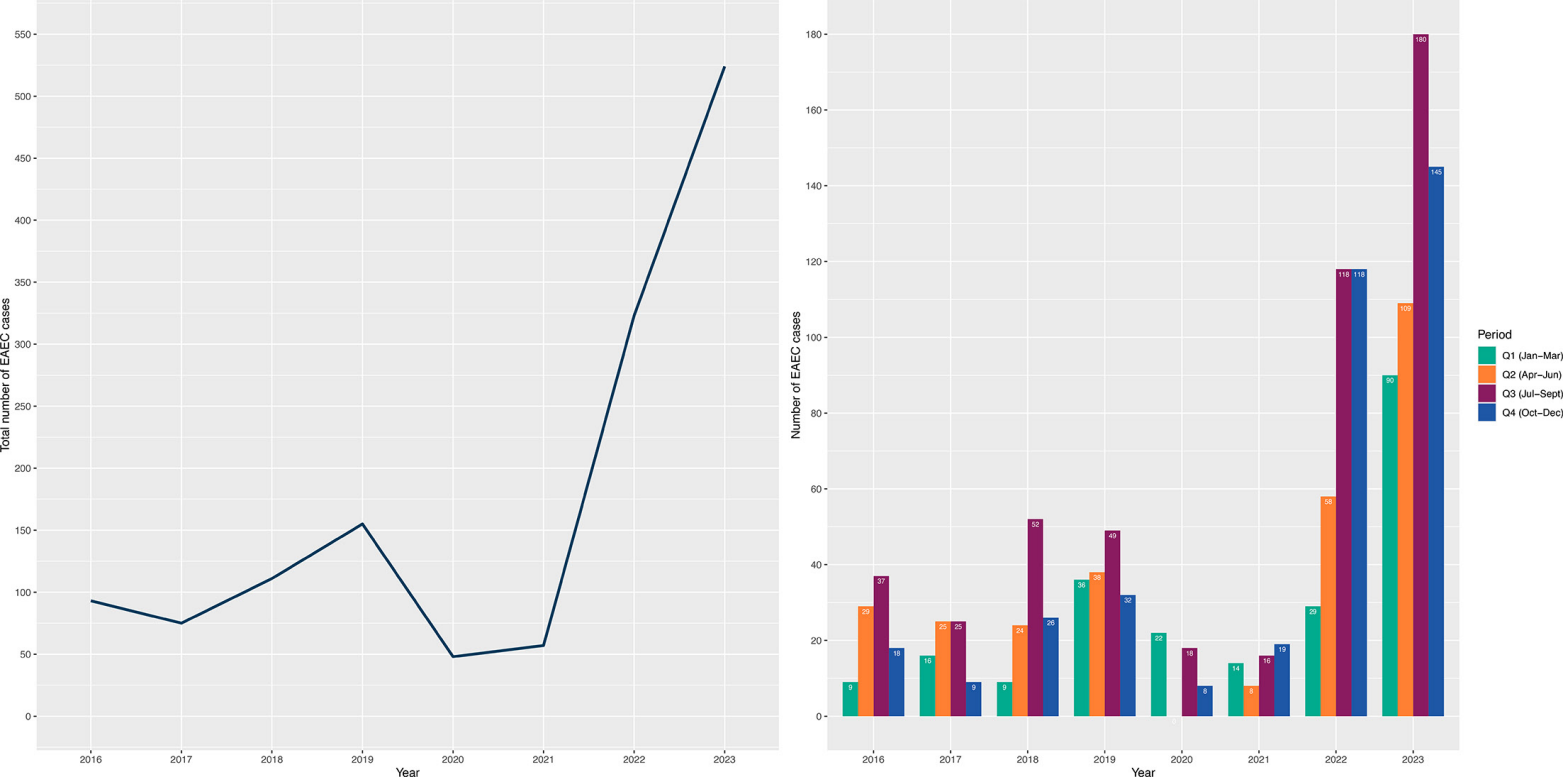
Number of EAEC cases referred to the GBRU between January 2016 and December 2023. A solid blue line represents total annual cases. Coloured bars represent cases organized by different quarters per year.

In total, there have been 1,402 human isolates of EAEC originating from 1,386 patients presenting with symptoms of diarrhoeal illness in England submitted to the GBRU between January 2016 and December 2023. There has been a year-on-year increase in diagnoses of EAEC infection, with 93 confirmed cases in 2016, increasing 5-fold to 524 confirmed cases in 2023. The number of confirmed cases declined in 2020 (*n*=48) and 2021 (*n*=57), attributed to the social distancing and travel restrictions imposed due to the COVID-19 pandemic (Fig. 1).

**Fig. 2.**
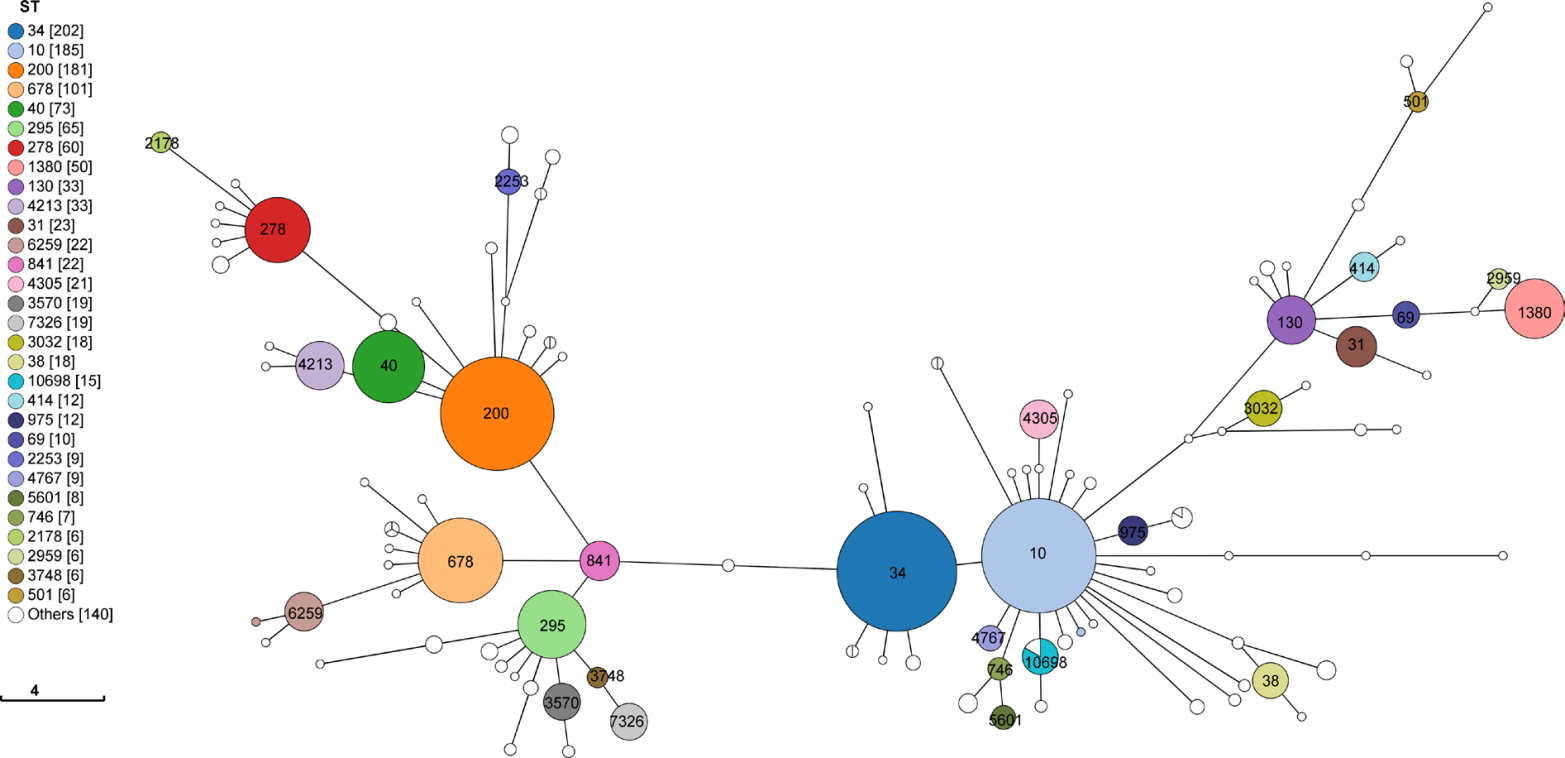
A 7-gene MLST minimal spanning tree of EAEC isolates with all identified STs annotated in this dataset.

Genome-derived MLST determined a total of 110 different STs, of which the top 10 STs were ST34 (*n*=202/1,402, 14.4%), ST10 (*n*=185/1,402, 13.2%), ST200 (*n*=183/1,402, 13.1%), ST678 (*n*=101/1,402, 7.2%), ST40 (*n*=74/1,402, 5.3%), ST295 (*n*=65/1,402, 4.6%), ST278 (*n*=60/1,402, 4.3%), ST1380 (*n*=50/1,402, 3.6%), ST130 (*n*=33/1,402, 2.4%) and ST4213 (*n*=33/1,402, 2.4%) (Fig. 2, Table 1). Genome-derived serotyping identified a total of 135 different serotypes of which the most common serotypes were O92:H33 (*n*=130/1,402, 9.3%), O175:H31 (*n*=78/1,402, 5.6%) and O99:H10 (*n*=78/1,402, 5.6%) (Table 1).

**Table 1.** Diversity of serotypes and STs of EAEC (*n*=1402)

Serotype	ST34	ST10	ST200	ST678	ST40	ST295	ST278	ST1380	ST130	ST4213	Other STs	Total
**O92:H33**	125	–	–	–	–	–	–	–	–	–	5	**130**
**O175:H31**	–	–	74	–	–	–	–	–	–	–	4	**78**
**O99:H10**	77	–	–	–	–	–	–	–	–	–	1	**78**
**O126:H27**	–	–	57	–	–	–	–	–	–	–	7	**64**
**O111:H21**	–	–	–	–	62	–	–	–	–	–	–	**62**
**O unidentifiable:H10**	–	–	–	–	–	36	–	–	–	–	23	**59**
**O181:H4**	–	–	–	45	–	–	4	–	–	–	3	**52**
**O104:H4**	–	–	–	44	–	–	1	–	–	–	3	**48**
**O44:H18**	–	–	–	–	–	–	–	29	–	–	19	**48**
**O176:H33**	–	19	–	–	–	–	–	–	–	–	21	**40**
**O3:H2**	–	39	–	–	–	–	–	–	–	–	–	**39**
**O55:H21**	–	–	–	–	–	–	–	–	–	33	2	**35**
**O unidentifiable:H19**	–	–	–	–	–	–	13	–	–	–	20	**33**
**O176:H34**	–	–	–	–	–	–	–	–	30	–	1	**31**
**O175:H28**	–	–	29	–	–	–	–	–	–	–	1	**30**
**O86:H2**	–	27	–	–	–	–	–	–	–	–	2	**29**
**O65:H12**	–	–	–	–	–	–	–	–	–	–	22	**22**
**O130:H27**	–	–	–	–	–	–	–	–	–	–	21	**21**
**O181:H16**	–	–	–	–	–	–	–	–	–	–	21	**21**
**O6:H10**	–	–	–	–	–	–	–	–	–	–	21	**21**
**O175:H27**	–	–	18	–	–	–	–	–	–	–	1	**19**
**O78:H10**	–	1	–	–	–	–	–	–	–	–	18	**19**
**O99:H4**	–	12	–	–	–	–	–	–	–	–	4	**16**
**O unidentifiable:H31**	–	–	–	–	–	–	–	–	–	–	15	**15**
**O131:H27**	–	–	–	–	–	13	–	–	–	–	–	**13**
**O33:H16**	–	–	–	–	–	13	–	–	–	–	–	**13**
**O114:H10**	–	11	–	–	–	–	–	–	–	–	1	**12**
**O86:H27**	–	–	–	–	–	–	–	–	–	–	12	**12**
**O unidentifiable:H4**	–	–	–	1	–	–	8	–	–	–	2	**11**
**O15:H18**	–	–	–	–	–	–	–	–	–	–	11	**11**
**O78:H2**	–	10	–	–	–	–	–	–	–	–	1	**11**
**O unidentifiable:H30**	–	1	–	–	–	–	–	–	–	–	9	**10**
**O131:H4**	–	–	–	3	–	–	7	–	–	–	–	**10**
**Other serotypes**	–	42	5	8	12	3	27	21	3	–	145	**266**
**Total**	**202**	**185**	**183**	**101**	**74**	**65**	**60**	**50**	**33**	**33**	**416**	**1,402**

Overall, ST34 (CC10) was the largest ST (*n*=202), mostly comprising two serotypes, O92:H33 (*n*=125/202, 61.9%) and O99:H10 (*n*=77/202, 38.1%). ST10 (CC10) (*n*=185) was more diverse, comprising 33 different serotypes, with the most common serotypes being O3:H2 (*n*=39/185, 21.1%) and O86:H2 (*n*=27/185, 14.6%). ST200 (CC40) (*n*=183) was comprised of seven distinct serotypes, of which O175:H31 was the most common (*n*=74/183, 40.4%), followed by O126:H27 (*n*=57/183, 31.1%). ST678 (*n*=101) was comprised of six distinct serotypes, of which O181:H4 (*n*=45/101, 44.6%) and O104:H4 (*n*=44/101, 43.6%) were the most common serotypes (Table 1).

**Fig. 3.**
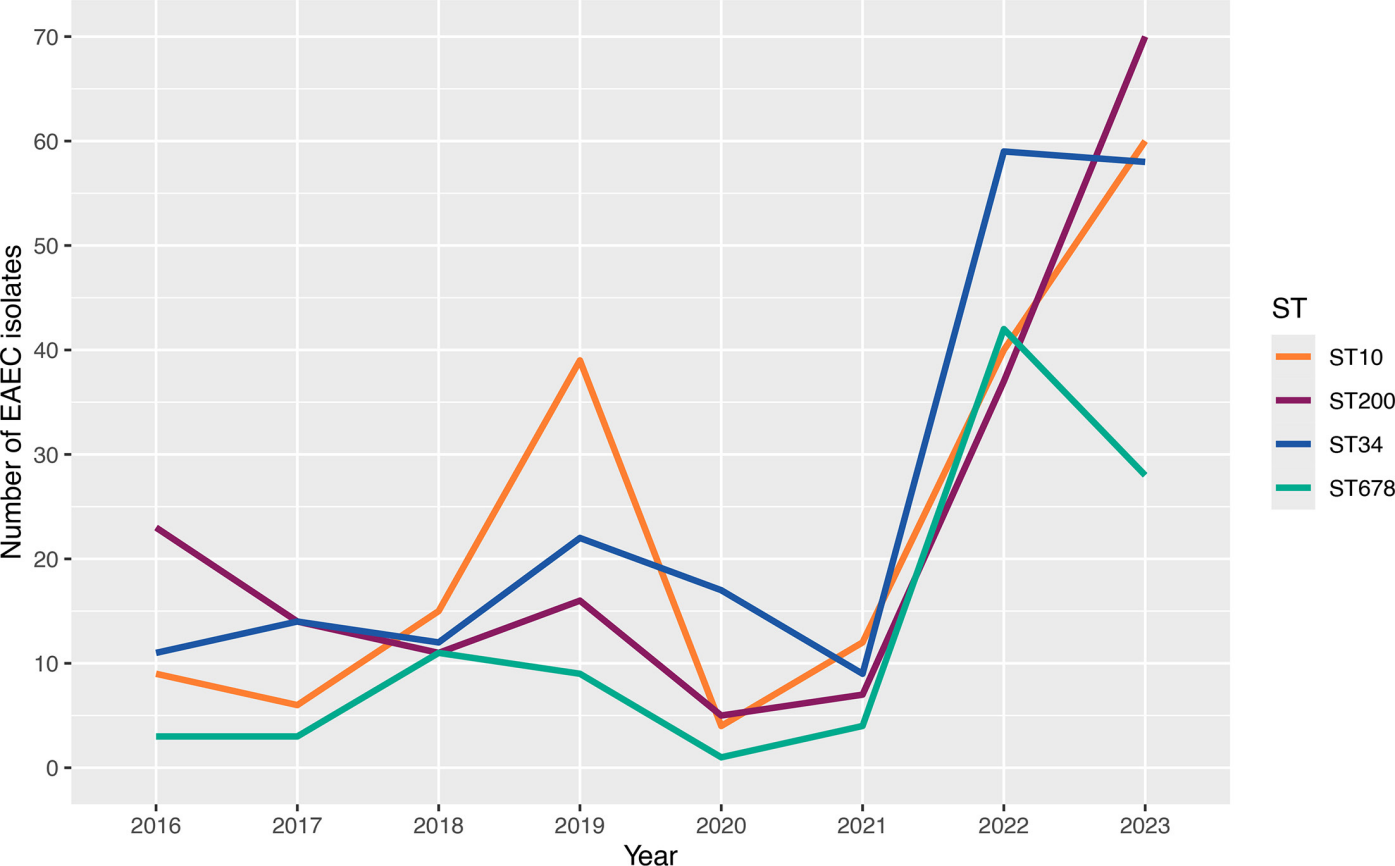
Annual isolate numbers belonging to the top four STs (ST34, ST10, ST200 and ST678) based on sample receipt date between 2016 and 2023.

The number of EAEC isolates associated with the top four STs fluctuated over the years but had a general increasing trend, with ST200 dominant in 2016 (*n*=23) and 2023 (*n*=70). ST10 notably peaked in 2019 (*n*=39) and 2023 (*n*=60), while ST34 and ST678 were dominant in 2022 (ST34 *n*=59; ST678 *n*=42) (Fig. 3). On average, the number of isolates associated with the top four STs increased nearly fivefold from 2016 (*n*=12) to 2023 (*n*=54).

### Descriptive epidemiology

**Fig. 4.**
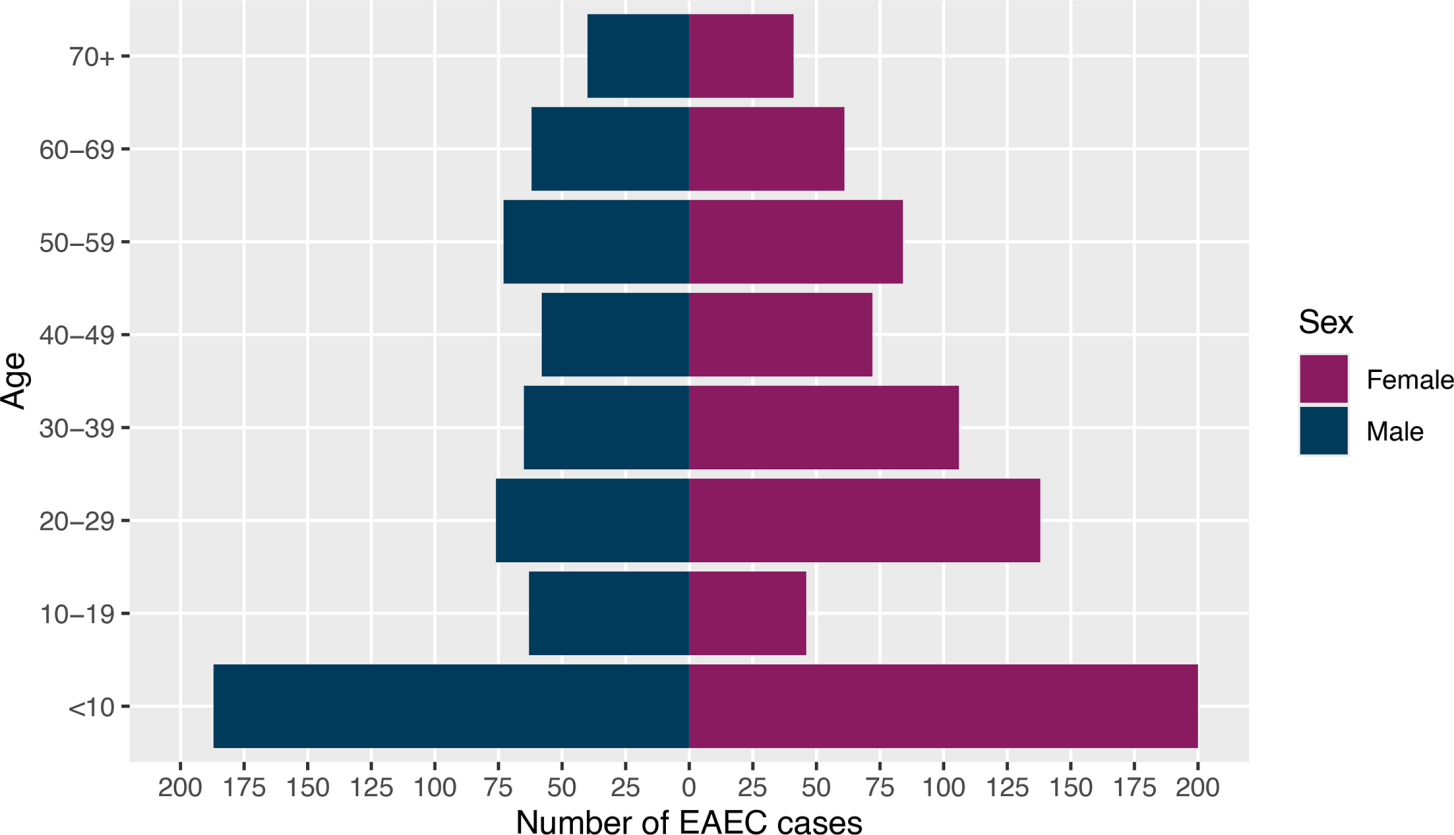
Age–sex distribution of total EAEC cases reported to UKHSA [isolates originating from England (*n*=1372), where date of birth, sample date and/or sample receipt date and sex data were available]. Blue indicates male data, while purple indicates female data.

The age–sex distribution of all EAEC cases, where age and sex data were available (*n*=1,372), indicated that the majority of cases were female (*n*=748/1,372, 54.5%), compared to male cases (*n*=624/1,372, 45.5%). The median age for females was 29 years (IQR: 8–49), while the median age for males was 28 years (IQR: 6–52). The ratio of female to male cases was equal or higher in nearly every age group, except for the 10 to 19 age group, where male cases were dominant (*n*=63/1,372, 4.6%) compared to female cases (*n*=46/1,372, 3.4%) (Fig. 4).

The highest proportion of cases belonged to the <10 age group for males and females combined (*n*=387/1,372, 28.2%), males only (*n*=187/1,372, 13.6%) and females only (*n*=200/1,372, 14.6%). Within the <10 age group, 299 out of 387 (77.3%) cases were <5 years of age (males: *n*=145, females: *n*=154). The second highest proportion of cases was observed in the 20 to 29 age group (*n*=214/1,372, 15.6%), where the incidence of female cases was almost twofold greater than male cases (females: *n*=138; males: *n*=76). The lowest proportion of cases was observed in the 70+ age group (*n*=81/1,372, 5.9%) (Fig. 4).

**Fig. 5.**
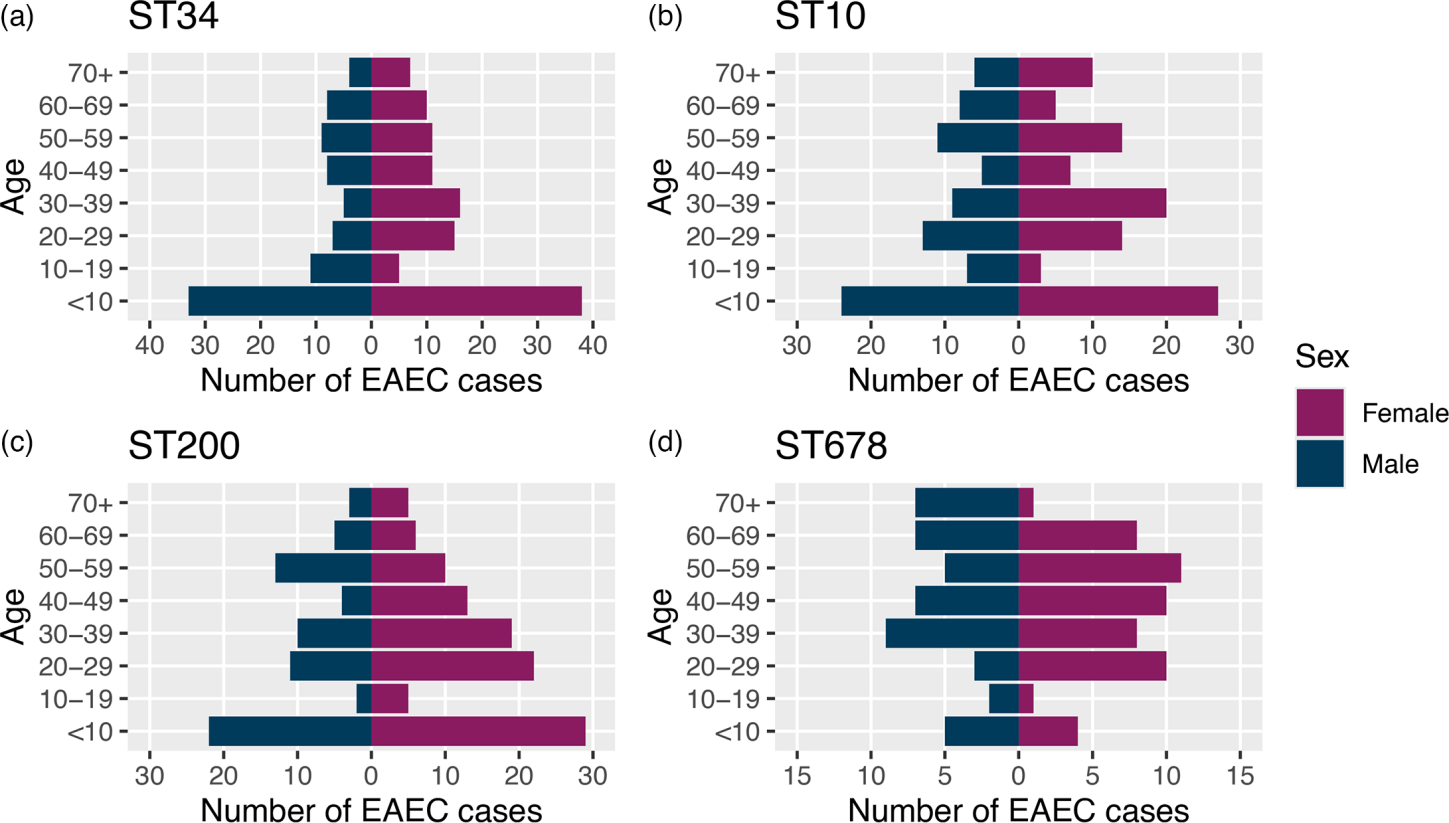
(a–d) Age–sex distribution of EAEC cases for the top four STs [ST 34 (*n*=198), ST10 (*n*=183), ST200 (*n*=179) and ST678 (*n*=98)] reported to UKHSA where date of birth, sample date and/or sample receipt date and sex data were available. Blue indicates male data, while purple indicates female data.

All of the top four STs for EAEC cases had a higher proportion of female cases (ST34 *n*=113/198, 57.1%; ST10 *n*=100/183, 54.6%; ST200 *n*=109/179, 60.9%; ST678 *n*=53/98, 54.1%). Cases were highest in children under 10 years of age (‘<10’) for ST34 (*n*=71/198, 35.9%), ST10 (*n*=51/183, 27.9%) and ST200 (*n*=51/179, 28.5%), indicating that cases in this age range were most at risk, while for ST678, cases were highest in adult patients aged between 30 and 49 (Fig. 5).

**Fig. 6.**
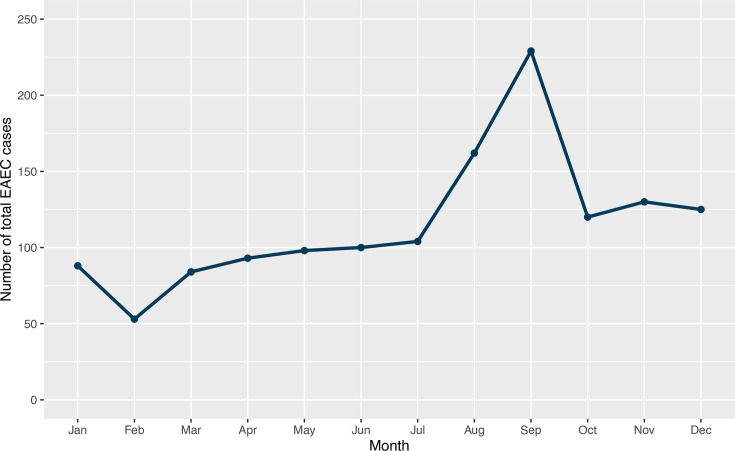
The number of EAEC cases (*n*=1386) by months between 2016 and 2023 to indicate seasonal variation in England.

Seasonal variation in the number of reported EAEC cases was observed, with a distinctive late summer peak in September (*n*=229), with lower levels during off-peak seasons from January to July (Fig. 6).

Based on available data (*n*=1,111), EAEC cases were detected in all regions of England, with the North of England having the highest frequency (*n*=363/1,111, 32.7%), followed by the South of England (*n*=337/1,111, 30.3%), the Midlands and East of England (*n*=235/1,111, 21.2%) and London (*n*=175/1,111, 15.8%), with cases typically clustered around major cities, notably London, Birmingham, Liverpool, Manchester, Newcastle and Leeds.

### Foreign travel

**Fig. 7.**
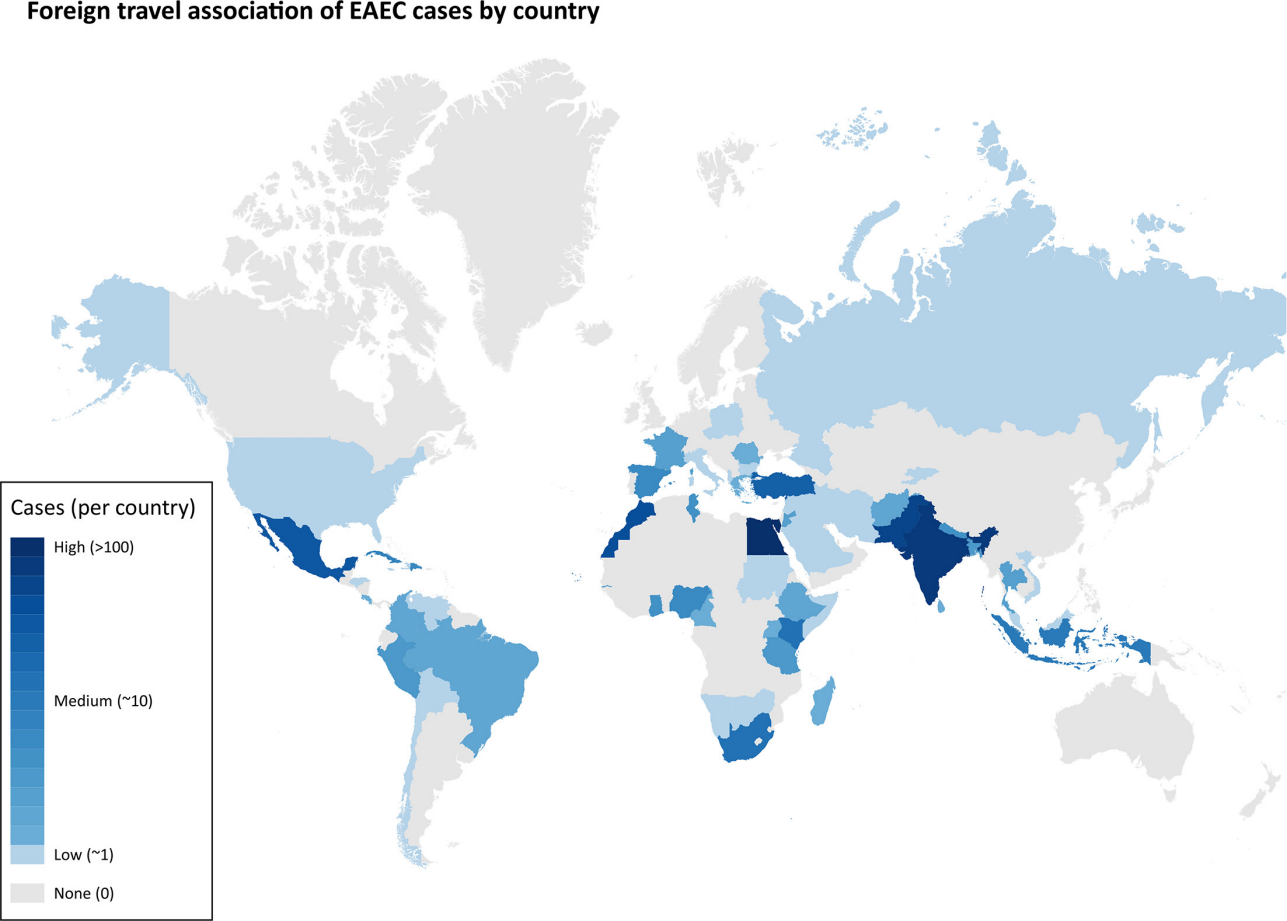
EAEC cases associated with foreign travel outside the UK, where a specific country was stated (*n*=535). Countries are colour-coded in proportion to the frequency of cases associated, i.e. the higher the frequency of cases, the darker the colour.

Of the 1,386 cases, 597 (43.1%) reported foreign travel in the 7 days prior to symptom onset, 159 (11.5%) were not associated with foreign travel and foreign travel status was unknown for 630 (45.5%) cases. The continental destinations most commonly reported were Africa (*n*=242, 17.5%) and Asia (*n*=219, 15.8%) (Table 2). The top five country destinations were Egypt (*n*=109), India (*n*=78), Pakistan (*n*=64), Morocco (*n*=38) and Mexico (*n*=36) (Fig. 7).

**Table 2.** Cases of EAEC reporting travel in 7 days prior to onset of symptoms, categorized by ST and continental travel destination

Continent	No. (%)				
	ST34 (*n*=202)	ST10 (*n*=184)	ST200 (*n*=181)	ST678 (*n*=100)	Total EAEC (*n*=1386)
Africa	24 (11.9)	35 (19.0)	25 (13.8)	43 (43.0)	242 (17.5)
Asia	24 (11.9)	19 (10.3)	23 (12.7)	12 (12.0)	219 (15.8)
Europe	4 (2.0)	5 (2.7)	2 (1.1)	0 (0.0)	25 (1.8)
North America	9 (4.5)	10 (5.4)	7 (3.9)	3 (3.0)	54 (3.9)
South America	8 (4.0)	4 (2.2)	3 (1.7)	0 (0.0)	23 (1.7)
Location not stated	5 (2.5)	5 (2.7)	3 (1.7)	5 (5.0)	34 (2.5)
Unknown	100 (49.5)	84 (45.7)	96 (53.0)	32 (32.0)	630 (45.5)
No travel	28 (13.9)	22 (12.0)	22 (12.2)	5 (5.0)	159 (11.5)
**Total**	**202 (100.0)**	**184 (100.0)**	**181 (100.0)**	**100 (100.0)**	**1,386 (100.0)**

Where travel was identified for cases belonging to the four most commonly detected STs, these were primarily associated with travel to Africa and Asia (Table 2). Egypt was the most common travel destination for all four STs (ST34 *n*=10, ST10 *n*=19, ST200 *n*=14 and ST678 *n*=14). For ST678, travel was predominantly associated with Africa (*n*=43/100, 43.0%), specifically Egypt (*n*=14) and Cape Verde (*n*=12).

### Genome-derived AMR profiles

**Fig. 8.**
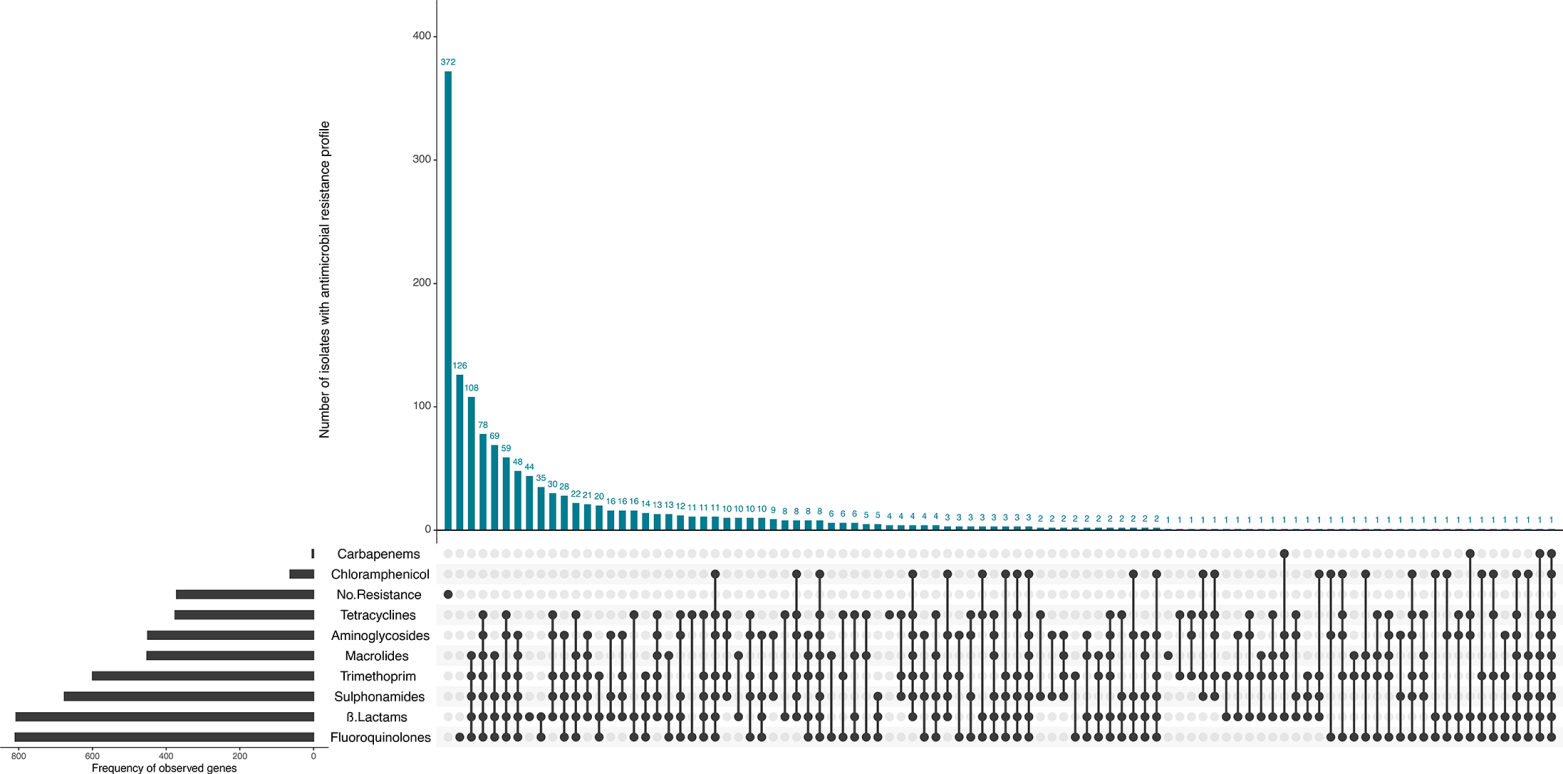
AMR profiles of EAEC isolates (*n*=1402). *In silico* detection of AMR genes was performed using GeneFinder and the UKHSA AMR gene database. AMR profiles are displayed using UpSetR.

Of all 1,402 isolates profiled *in silico*, 372 out of 1,402 (26.5%) did not have any AMR determinants in the UKHSA GeneFinder reference database, and susceptibility to all 9 antimicrobial classes included in this analysis was inferred (Fig. 8). There were 1,030 out of 1,402 (73.5%) isolates that had AMR determinants known to confer resistance to at least 1 antimicrobial class.

### Resistance to fluoroquinolones and macrolides

AMR determinants known to confer reduced susceptibility or resistance to fluoroquinolones were present in 810 out of 1,402 (57.8%) isolates. Of these 1,402 isolates, mutations in the quinolone resistance-determining region (QRDR) of the *gyrA* and *parC* genes were detected in 588 out of 1,402 (41.9%) isolates. The most common QRDR mutations included *gyrA*[83:S-L] (*n*=305) and *gyrA*[83:S-A] (*n*=165). Plasmid-mediated quinolone resistance determinants were detected in 398 out of 1,402 (28.4%) isolates, where *qnrB4* (*n*=241) and *qnrS1* (*n*=107) were the most frequently detected.

There were 452 out of 1,402 (32.2%) isolates with AMR determinants associated with macrolide resistance. The majority of isolates with predicted macrolide resistance harboured *mph(A*) (*n*=449), followed by *erm(B*) (*n*=25).

### Resistance to β-lactams and carbapenems

Out of 1,402 isolates, 807 (57.6%) harboured AMR determinants known to confer resistance to the β-lactams. The most detected AMR determinants encoded the penicillinase *bla_TEM-1_* (*n*=463), acquired *AmpC* gene *bla_DHA-1_* (*n*=240) and extended-spectrum β-lactamase (ESBL) *bla_CTX-M-15_* (*n*=212). Carbapenemase genes were also detected in four isolates: *bla*_OXA-48_ (*n*=1), *bla*_OXA-181_ (*n*=1), *bla*_OXA-244_ (*n*=1) and *bla*_NDM-5_ (*n*=1).

### Resistance to folate pathway antagonists

Out of 1,402 isolates, 600 (42.8%) had *dfrA* variants conferring resistance towards trimethoprim, of which *dfrA17* (*n*=220) was the most common, followed by *dfrA1* (*n*=123) and *dfrA7* (*n*=108). There were 676 (48.2%) isolates which had genes predicted to confer resistance to sulphonamides, where *sul1* (*n*=423) was the most prevalent, followed by *sul2* (*n*=349).

### Resistance to aminoglycosides

There were 450 out of 1,402 (32.1%) isolates detected to harbour AMR determinants known to confer resistance to the aminoglycosides. The combined profile *strA-strB* conferring resistance to streptomycin was present in 290 isolates, while *aadA* variants conferring resistance to both spectinomycin and streptomycin were detected in 166 isolates, of which *aadA5* (*n*=80) and *aadA1* (*n*=55) were the most common.

### Resistance to tetracyclines and phenicols

Out of 1,402 isolates, 376 (26.8%) harboured AMR determinants known to confer resistance to the tetracyclines. These consisted primarily of *tet(A*) (*n*=362), followed by *tet(D*) (*n*=13) and *tet(B*) (*n*=2).

Sixty-four (4.6%) isolates harboured AMR determinants predicted to confer resistance to chloramphenicol and/or florfenicol, where *catA1* (*n*=56) was detected the most frequently. Other AMR determinants include *catB3* (*n*=3), *catB7* (*n*=2), cml1* (*n*=3); and *floR* (*n*=4).

### Multi-drug resistance

There were 777 out of 1,402 (55.4%) isolates which harboured AMR determinants conferring resistance against 3 or more antimicrobial classes and were categorized as multi-drug-resistant (MDR). Of these 777 MDR isolates, 379 (48.8%) were associated with foreign travel, with the most common travel destinations being Egypt (*n*=81), Pakistan (*n*=53) and India (*n*=52). Seventy-three (9.4%) isolates had no travel association, while travel association was unknown for 325 (41.8%) isolates.

The most common resistance profile was AMR determinants associated with fluoroquinolone resistance (*n*=126/1,402, 9.0%); followed by macrolides, trimethoprim, sulphonamides, β-lactams and fluoroquinolone resistance (*n*=108/1,402, 7.7%); and tetracyclines, aminoglycosides, macrolides, trimethoprim, sulphonamides, β-lactams and fluoroquinolone resistance (*n*=78/1,402, 5.6%) (Fig. 7).

For ST34, the proportion of sensitive isolates (*n*=97/202, 48.0%) to resistant isolates (*n*=105/202, 52.0%) was comparable, while ST10 and ST200 had a higher ratio of resistant isolates (ST10 *n*=140/185, 75.7%; ST200 *n*=110/183, 60.1%). All ST678 isolates had AMR determinants predicted to confer resistance to at least one antimicrobial class. Each of the four STs had a high proportion of MDR isolates (ST34 *n*=78/105, 74.3%; ST10 *n*=103/140, 73.6%; ST200 *n*=96/110, 87.3%; ST678 *n*=93/101, 92.1%) (Table 3).

**Table 3.** EAEC isolates belonging to ST34, ST10, ST200 and ST678 categorized as either sensitive or resistant, and their association with foreign travel

Isolates	ST34 (*n*=202)	ST10 (*n*=185)	ST200 (*n*=183)	ST678 (*n*=101)
**Sensitive**	**97 (48.0)**	**45 (24.3)**	**73 (39.9)**	**0 (0.0)**
No travel	15 (15.5)	4 (8.9)	14 (19.2)	0 (0.0)
Travel	28 (28.9)	16 (35.6)	45 (61.6)	0 (0.0)
Travel unknown	54 (55.7)	25 (55.6)	14 (19.2)	0 (0.0)
**Resistant**	**105 (52.0)**	**140 (75.7)**	**110 (60.1)**	**101 (100.0)**
No travel	13 (12.4)	18 (12.9)	8 (7.3)	5 (5.0)
Travel	46 (43.8)	62 (44.3)	53 (48.2)	64 (63.4)
Travel unknown	46 (43.8)	60 (42.9)	49 (44.5)	32 (31.7)
**MDR**	**78 (74.3)**	**103 (73.6)**	**96 (87.3)**	**93 (92.1)**

Where foreign travel was indicated, sensitive isolates were most commonly associated with travel to Africa (ST34 *n*=17; ST10 *n*=11; ST200 *n*=8), while AMR isolates were associated with travel to both Africa (ST34 *n*=7; ST10 *n*=23; ST200 *n*=17; ST678 *n*=44) and Asia (ST34 *n*=21; ST10 *n*=19; ST200 *n*=19; ST678 *n*=12).

### Hybrid EAEC-STEC

During this time frame of this study, we identified six isolates of EAEC that had the Shiga toxin gene (*stx*) (Table 4). Three isolates belonged to the same ST (ST678) as the aetiological agent that caused the outbreak of HUS in Germany in 2011, and one belonged to the same serotype, O104:H4. None of the cases infected with these EAEC-STEC hybrid strains developed HUS.

**Table 4.** Hybrid STEC/EAEC isolates harbouring *stx2* and *aggR* detected between 2016 and 2023

SRA accession	Year	Travel	Serotype	ST	Stx subtype
SRR6489773	2018	India	O181:H4	678	stx2a
SRR14071634	2021	No data	O175:H31	200	stx2a
SRR22270452	2022	Cape Verde	O181:H4	678	stx2a
SRR29077551	2023	No data	O104:H4	678	stx2a

## Discussion

Molecular diagnostic assays for the detection of STEC in faecal specimens have been in use at GBRU since 1990; however, following the outbreak of STEC-EAEC O104:H4 in Germany in 2011, we took steps to widen our surveillance of diarrhoeagenic *E. coli* (DEC) to include EAEC. Initially, the number of EAEC detected each year remained relatively low compared to STEC. Like other gastrointestinal pathogens, notifications decreased during the COVID-19 pandemic, before rapidly increasing once lockdown restrictions were lifted [39]. The cause of the notable increase in EAEC diagnoses is unclear but may have been driven by a combination of more widespread molecular testing for GI pathogens in the UK, a resurgence of UK residents travelling abroad after the travel restrictions implemented during the COVID-19 pandemic were lifted, reduced immunity to enteric pathogens in children under the age of five as a result of social distancing measures implemented during the COVID-19 pandemic and/or increasing antibiotic resistance.

Analysis of the EAEC population structure in this study revealed a diverse group belonging to a wide range of different CCs and STs exhibiting a variety of different serotypes within each ST [40]. This diversity is due to the defining feature of the EAEC pathotype, *aggR*, being located on a highly transmissible plasmid [41]. Nevertheless, certain STs and serotypes reported in this study have been consistently reported in global surveillance studies [42]. Commonly detected EAEC serotypes in this study, specifically O99:H10, O92:H33 and O111:H21, have caused outbreaks in Japan, Italy and Belgium, respectively [43,45].

Multiple serotypes of EAEC belonging to ST678 have caused large foodborne outbreaks, including EAEC O104:H4 in Germany and elsewhere in 2011 [29,31] and EAEC O181:H4 in England in 2014 [20]. The 2011 German outbreak of EAEC O104:H4 was epidemiologically linked to contaminated fenugreek seeds [30]. The outbreak in the UK in 2011 caused by multiple pathogens EAEC O181:H4 involved over 400 people, reporting symptoms commonly associated with EAEC infection, specifically abdominal cramps and persistent diarrhoea [20,25]. Risk factors associated with illness included eating foods from one particular vendor and eating a food item containing uncooked curry leaves. Strains of EAEC were detected in the food handlers, and contamination of the food by the food handlers was thought to be the most likely source [20,25]. Other foodborne outbreaks caused by EAEC have been reported in Japan [21], the UK [24] and South Korea [23], all thought to be caused by infected food handlers. The infection status of food handlers, including asymptomatic carriage of EAEC, and hygienic conditions applied during the handling and processing of foodstuffs in some countries appear to be an important factor in contamination of foods at retail, catering or household level [46].

Previous studies, mainly in Africa, identified EAEC in a high proportion of children with diarrhoea and linked colonization with morbidity and malnutrition [1,4,6]. In this study, we observed the highest proportion of EAEC in children, although that may reflect parental behaviours and public health guidance that prioritize gastrointestinal illness in children over adults. Parents are more likely to take their symptomatic child to hospital emergency departments or their general practitioner (GP) than to present themselves. GPs are more likely to refer faecal specimens from children presenting with gastrointestinal symptoms for microbiological analysis, and these specimens are more likely to be referred to GBRU for follow-up testing by the local hospital diagnostic laboratories, as children aged five and under are considered a vulnerable group [47,48]. In contrast to the majority of EAEC STs, ST678 was more commonly isolated in adults than children, and this may reflect the pathogenicity of the strain causing more severe symptoms or may indicate that this ST has a different transmission route. We identified a higher proportion of females than males, with similar ratios as we see in the UKHSA STEC surveillance data [49,50]. Historically, this has been attributed to the increased risk of exposure during food preparation and childcare, although these are no longer regarded as traditionally female roles.

EAEC is known to be a cause of travellers’ diarrhoea, and the analysis in our study provides further evidence of this association [51]. Of those cases reporting travel data, nearly 80% reported travelling outside the UK during weeks prior to onset of symptoms and just over 20% stating that they had not travelled. UKHSA and NaTHNaC offer advice to UK citizens travelling to regions with a high risk of gastrointestinal disease [52]. However, preventing infection while abroad is challenging, particularly in countries where the prevalence of EAEC is high. Transmission can occur via consumption of food contaminated by colonized food handlers, person-to-person contact and exposure to an environment, such as insufficiently chlorinated recreational water or an unhygienic toilet facility. Travel destinations associated with the highest number of cases were the Indian sub-continent, Egypt, Morocco and Mexico, providing evidence that EAEC is dispersed globally and that the incidence and prevalence are high in countries often associated with high rates of AMR [53].

DEC isolated from returning travellers is associated with multi-drug resistance, and EAEC have been shown to have the highest proportion of AMR determinants of all the DEC groups [2,3]. Symptoms associated with EAEC are generally mild and self-limiting but are often prolonged, typically lasting 10 days or more, and individuals may seek treatment if their symptoms fail to resolve. Ciprofloxacin was traditionally a first-line antibiotic used to treat traveller’s diarrhoea; however, resistance to fluoroquinolones has become an increasing problem in some parts of the world, and most treatment regimens now recommend azithromycin [52,54]. Although the third-generation cephalosporins are not routinely used to treat gastrointestinal infections, the detection of AMR determinants conferring resistance to ESBLs and carbapenemase antibiotics is a public health concern as these determinants are often encoded on mobile genetic elements and can be transmitted to commensal gut flora [55,56].

Although antibiotic treatment for EAEC infections is not recommended, monitoring AMR in this pathotype may be useful as a barometer for AMR providing a way to monitor the global threat of AMR and encourage collective action [57]. Recent studies have heightened the need to continue to improve our monitoring and understanding of infectious disease globally and ensure that the surveillance of drug-resistant infections is included in our surveillance systems [57]. UKHSA is committed to promoting the removal of barriers to safe, secure and appropriate sharing of data of use to global surveillance efforts.

Despite enhanced surveillance implemented in the aftermath of the outbreak of STEC-EAEC O104:H4 in Germany in 2011, there have been few notifications of hybrid STEC-EAEC strains in the UK or elsewhere [34,35]. However, as the Shiga toxin encoding determinant *stx* is located on a bacteriophage, and there is evidence to suggest that a wide range of *E. coli* are susceptible to this mobile genetic element, the risk of outbreaks of HUS caused by hybrid STEC-EAEC remains [58,61].

IID studies carried out in the UK in 1993–1996 and 2008–2009 revealed that EAEC were among the most commonly isolated bacterial pathogens in patients with symptoms of gastroenteritis [11,13, 62]. The third IID study is currently in progress and is proving further evidence that EAEC continues to cause a high burden of community-acquired IID (Microsoft Power BI). Given the burden of disease caused by EAEC in the community, the high proportion of infections in children, the risk of the emergence of hybrid pathotypes and the utility of UK travel data to inform surveillance of GI pathogens and act as a proxy for the surveillance incidence and prevalence of AMR in resource-limited settings, we concluded that EAEC should be part of the diagnostic algorithm in the UK. Surveillance strategies should include analysis of travel data and monitoring AMR to inform the global perspective.

## Supplementary material

10.1099/jmm.0.002097 Uncited Table S1.

## Abbreviations

AMR: antimicrobial resistance
DEC: diarrhoeagenic *Escherichia coli*
EAEC: enteroaggregative *Escherichia coli*
EIEC: enteroinvasive *Escherichia coli*
EPEC: enteropathogenic *Escherichia coli*
ESBL: extended-spectrum β-lactamase
ETEC: enterotoxigenic *Escherichia coli*
GBRU: Gastrointestinal Bacteria Reference Unit
GDW: Gastro Data Warehouse
GP: general practitioner
IID: infectious intestinal disease
MDR: multi-drug-resistant
QRDR: quinolone resistance-determining region
SRST: short-read sequence typing
STEC: Shiga toxin-producing *Escherichia coli*
STs: sequence types
TSB: tryptone soya broth
UKHSA: UK Health Security Agency
